# Dataset on leaf surface and elemental study of four species of Bignoniaceae family by SEM-EDAX

**DOI:** 10.1016/j.dib.2018.02.037

**Published:** 2018-02-17

**Authors:** Kalyani Abhimanyu Kedar, Sanjay Ravindra Chaudhari, Avanapu Srinivasa Rao

**Affiliations:** a Department of Pharmacognosy, Progressive Education society's Modern College of Pharmacy, Sector -21, Yamunanagar Nigdi, Pune 411044, Maharashtra, India; b Rasiklal M. Dhariwal Institute of Pharmaceutical Education and Research, Pune, India; c Bhaskar Pharmacy College, Yeknapally, Moinabad (Mandal), R.R(Dt), Hyderabad 500075, India; d Jawaharlal Nehru Technological University (JNTU), Hyderabad 500072, Andra Pradesh, India

**Keywords:** BSI, botanical survey of India, SEM-EDS, scanning electron microscopy- Energy dispersive spectroscopy, Bignoniaceae, *Tecoma*, *Tabebuia*, *Tecoma gaudichaudi* DC, *Tecoma capensis* (Thunb.) Lindl, *Tecoma stans* (L.) Juss.ex Kunth, *Tabebuia rosea* (Bertol), Scanning electron microscopy, Elemental analysis

## Abstract

The data presented in this article are related to the scanning electron microscope and elemental studies in the four species of Bignoniaceae namely *Tecoma gaudichaudi* DC (Sample 1), *Tecoma capensis* (Thunb.) Lindl. (Sample 2), *Tecoma stans* (L.) Juss.Ex Kunth (Sample 3), *Tabebuia rosea* (Bertol.) (Sample 4). The SEM images were obtained for permanent record. The abaxial and adaxial surfaces of each species were carefully studied. In addition to this, the consistent occurrence of anomocytic stomata in all four species of this family shows that morphological and taxonomically all the species are very close and intimate.

The elemental data on leaf samples of all four species were performed and total eight important components were present such as C, O, Mg, Al, Si, Cl, K, Ca. These elements are useful, so identification of inorganic components of these species defiantly helps to promote as dietary elements.

**Specifications Table**

**Table:** 

**Subject area**	*Plant science*
**More specific subject area**	*Morphological and elemental analysis of species of Bignoniaceae Family.*
**Type of data**	*SEM micrograph of leaf surface, SEM-EDS (Scanning electron microscopy- Energy dispersive spectroscopy)*
**How the data were acquired**	*FEI (Field Emission Ion) -QUANTA 200 with EDS (3.0.13) with microscope Control Software.*
**Data format**	*SEM micrograph and Scanning electron microscopy- Energy dispersive spectroscopy spectra*
**Experimental factors**	*Fresh leaf samples of Tecoma gaudichaudi DC (Sample 1), Tecoma capensis (Thunb.) Lindl. (Sample 2), Tecoma stans (L.) Juss.* Ex *Kunth (Sample 3), Tabebuia rosea (Bertol.) (Sample 4)*
**Experimental features**	*The freshly collected leaf samples and its powder of all species of the Bignoniaceae family (Tecoma gaudichaudi* DC*, Tecoma capensis (Thunb.) Lindl., Tecoma stans (L.) Juss.ex Kunth, Tabebuia rosea (Bertol.)) was observed by the FEI (Field Emission Ion) -QUANTA 200 with EDS (3.0.13) with microscope Control Software and mode of operation were varied with low vacuum, high vacuum, ESEM. During SEM study various parameters were monitored such as Voltage range (200 V–30 KV), Magnification (30X–300,000x), Pressure Range is in between 10 Pa*–*130 Pa (Low vacuum), for this study pressure range was near to 65 Pa.*
**Data source location**	*Department of Pharmacognosy,*
	*Progressive Education Society's Modern College of Pharmacy,*
	*Sector -21, Yamunanagar Nigdi, Pune-411044, Maharashtra*
**Data accessibility**	*All data are given along with the article and will be available for education and research work.*

**Value of the data**

■ The data present scanning electron microscopy images and elemental investigation of four species of the Bignoniaceae namely *Tecoma gaudichaudi* DC, *Tecoma capensis* (Thunb.) Lindl, *Tecoma Stans* (L.) Juss. Ex Kunth, *Tabebuia rosea* (Bertol.).
■ Data represents SEM images and EDS spectra for permanent record.
■ This data helps to other researchers for the authentication of all the four species.
■ The current data on inorganic components defiantly help to promote these species as dietary elements.

## 1 Data

The present data focus on scanning electron microscopy and elemental testing of *Tecoma* and *Tabebuia* genus by SEM EDAX. *Bignonia* Linn (Bignoniaceae) is a monotypic genus of woody climbers, native to North America and mostly grown for ornament in the tropics of the old world [1]. Bignoniaceae family having 100 genera and more than 750 plant species found in various tropical regions of India and other country. The Fischer et al. [2] Reports recent classification of Bignoniaceae family and identify the seven tribes such as Bignonieae, Coleeae, Crescentieae (Tabebuia), Eccremocarpeae, Oroxyleae, Tacoma, and Tourrettieae. These are observed as succulent herbs, shrubs, stem sometimes reduced to a rhizome or tuber. Great diversity observes in the Bignoniaceae family in leaf, floral morphology, flowering phenology, seasonality etc. [3]. The data of scanning electron microscopy of the various species of the Bignoniaceae family show presence of anomocytic stomata on lower leaf surface with a stone microscopic pore on the leaf epidermal surface in addition with patelliform trichomes commonly [4], [5], [6]. Taking these recent studies into account, various species of this family poorly known to the African and Asian groups. The present finding helps in the identification of individual plant species which collected from different regions of Maharashtra State.

## 2 Experimental design, materials and methods

### 2.1 Collection and identification of plant species

All four species of Bignoniaceae family, i.e. *Tecoma gaudichaudi* DC (Sample 1), *Tecoma capensis* (Thunb.) Lindl. (Sample 2), *Tecoma stans* (L.) Juss. Ex Kunth (Sample 3), *Tabebuia rosea* (Bertol.) (Sample 4) was collected from different area of Pune district (Maharashtra) and the plant were authenticated at the Botanical Survey of India (BSI), Pune with reference no. BSI/WRC/Iden. /2015/576 on dated 18-12-2015. The specimen voucher number is KALKTEG1, KKA-2, KKA-3, KKA-1. Specimens of plants were deposited at the Botanical Survey of India, Pune and department of Pharmacogosy, Modern College of Pharmacy, Nigdi, Pune.

### 2.2 A leaf surface study by scanning electron microscopy

**Fig. 1:**
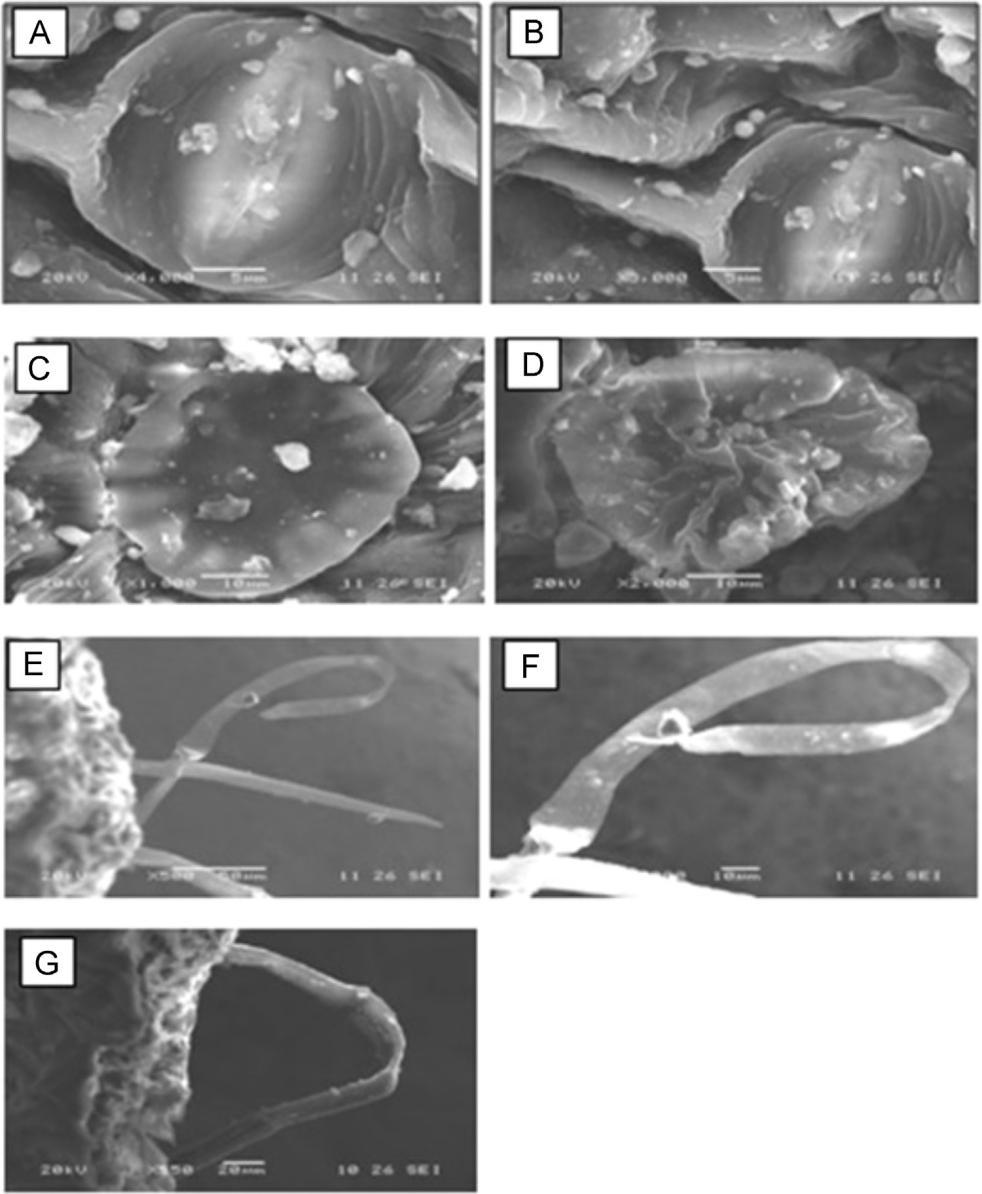
SEM micrograph of leaf surface of *Tecoma gaudichaudi* DC (A–B) *Tecoma gaudichaudi* (abaxial surface) showing closed anomocytic stomata, (C-D) *Tecoma gaudichaudi* DC (abaxial surface) showing patelliform trichomes, (E–F) *Tecoma gaudichaudi* (abaxial surface) showing covering trichomes, (G) *Tecoma gaudichaudi* (adaxial surface) surface showing covering trichomes.

**Fig. 2:**
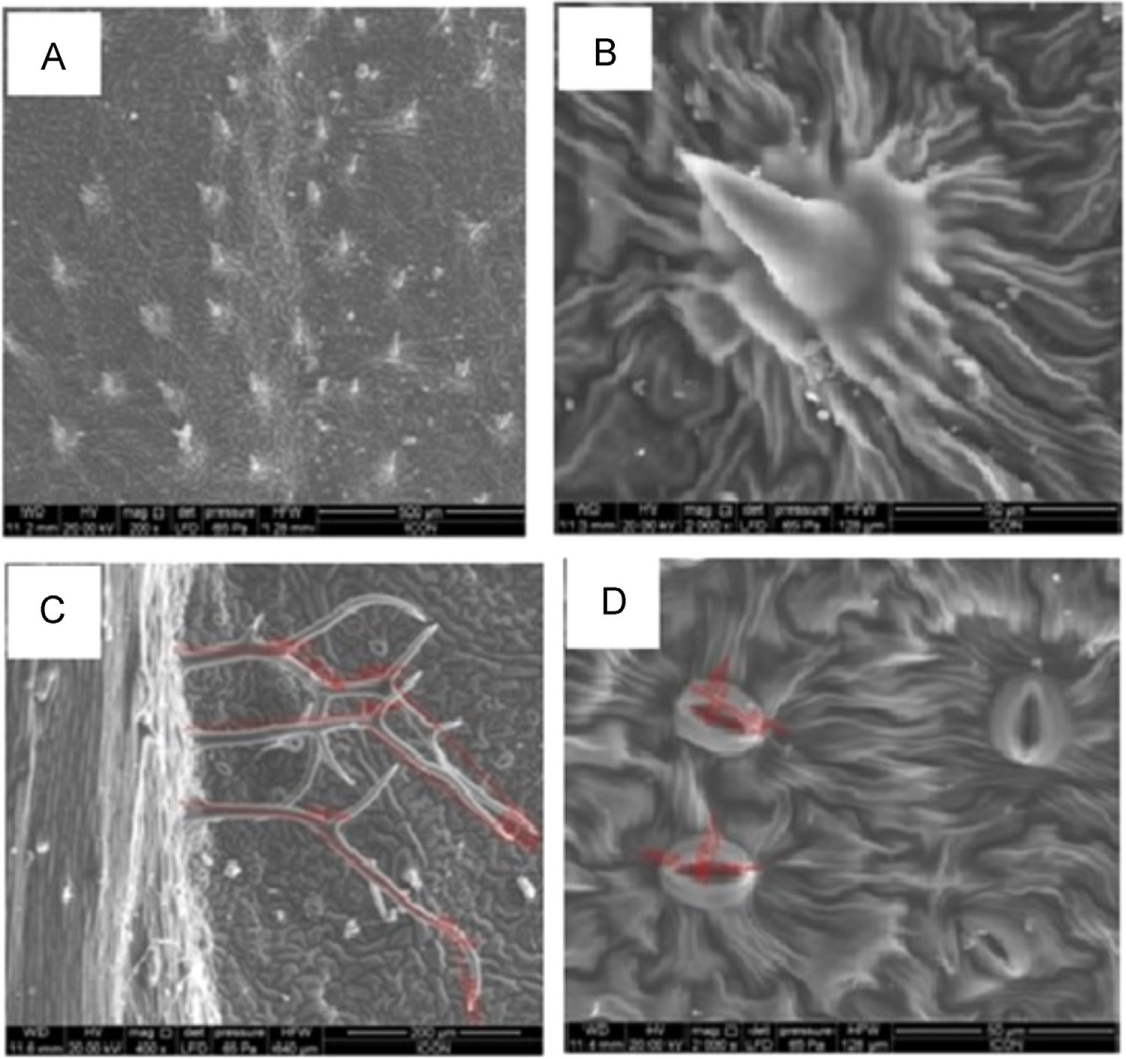
Scanning electron micrograph of the leaves of *Tecoma capensis* (A-B) *Tecoma capensis* (adaxial) showing unicellular trichomes with wavy wall base, (C-D) *Tecoma capensis* (abaxial) branched trichomes (average length 470.63 µm) and raised stomata (average diameter 16.51 µm).

**Fig. 3:**
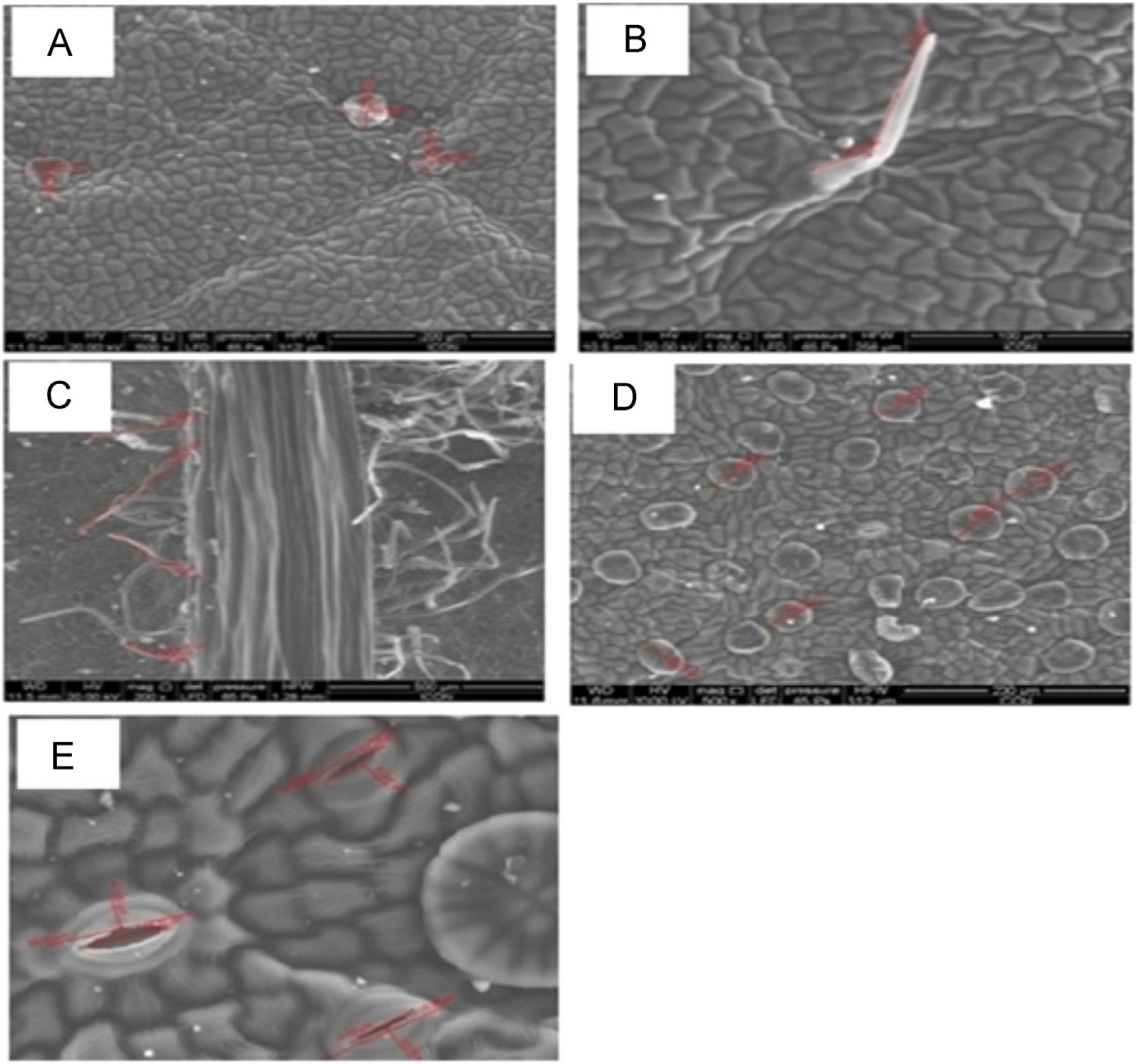
Scanning electron micrograph of the leaves of *Tecoma stans* (L.) Juss. Ex Kunth (A-B) *Tecoma Stans* (L.) adaxial leaf surface showing glandular/patelliform trichomes (diameter 45.26 µm), non glandular covering trichomes very few in number, (C) *Tecoma stans* abaxial leaf surface showing multicellular (1–5cells; average length 244.27 µm), (D) *Tecoma stans* (L.) abaxial leaf surface showing the patelliform type of trichomes (average diameter 46.86 µm), (E) *Tecoma Stans* abaxial leaf surface shows raised anomocytic stomata (average diameter 16.30 µm).

**Fig. 4:**
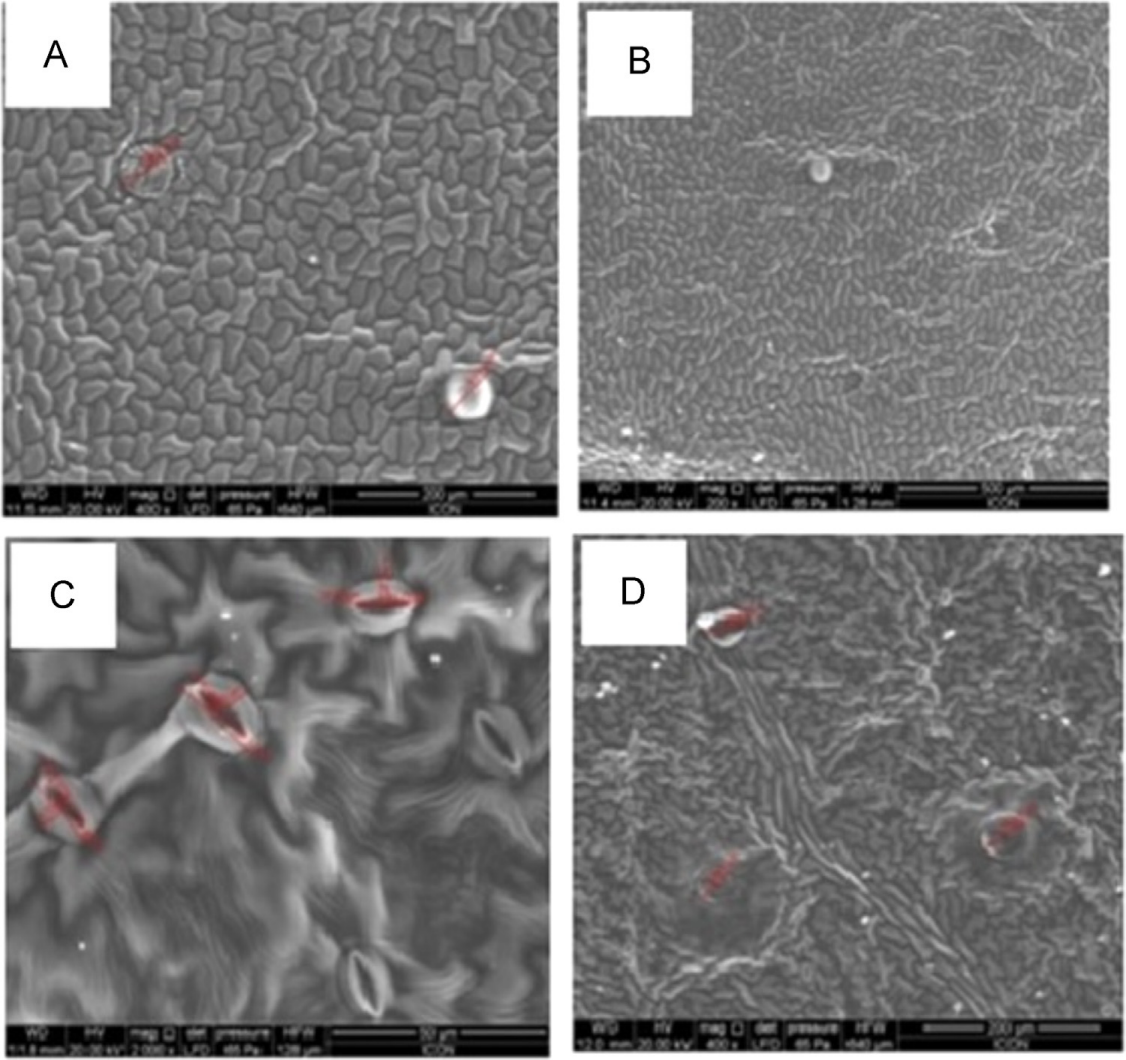
Scanning electron micrograph of the leaves of *Tabebuia rosea* (Bertol.) (A-B) *Tabebuia rosea* (Bertol.) (adaxial leaf surface) shows patelliform trichomes (average diameter 60.82 µm), (C-D) *Tabebuia rosea* (Bertol.) (abaxial leaf surface) showing raised anomocytic stomata (diameter 12.30 µm), patelliform trichome (diameter 46.81 µm).

Freshly collected leaf samples (1, 2, 3 and 4) were observed on both sides by the FEI (Field Emission Ion) -QUANTA 200 with EDS (3.0.13) with microscope Control Software and mode of operation were varied with low vacuum, high vacuum, ESEM. During SEM study various parameters were monitored such as such, as the voltage range (200 V to 30 KV), magnification (30X–300,000x), pressure range (10–130 Pa). (Fig. 1, Fig. 2, Fig. 3, Fig. 4).

Current data by scanning electron microscopy help in the recognition of *Tecoma gaudichaudi* DC and *Tecoma stans* (L.) Juss. ex Kunth. Data also helps in detection of *Tecoma capensis* (Thunb.) Lindl as compared with other species. So current data reveals that all the species of the Bignoniaceae family confirm the presence of the anomocytic kind of stomata on abaxial leaf surface, this was a very distinctive character observed regarding *Tecoma* species (Table 1). The consistent occurrence of anomocytic stomata in all four species of this family shows that morphological and taxonomically all the species are very close and intimate.

**Table 1:** Qualitative leaf anatomical characters of the species of Bignoniaceae family.

**Name of species (Family Bignoniaceae)**	**Types of trichomes Covering/multicellular/Branched/patelliform or glandular**		**Type of Stomata**	
	**Adaxial leaf surface**	**Abaxial surface of leaf**	**Adaxial surface of leaf**	**Abaxial surface of leaf**
*Tecoma gaudichaudi* DC	Multicellular and Patelliform	Multicellular and Patelliform	–	Anomocytic
*Tecoma capensis* (Thunb.) Lindl	Unicellular	Branched	–	Anomocytic
*Tecoma stans* (L.) Juss.ex Kunth	Patelliform	Multicellular	–	Anomocytic
*Tabebuia rosea* (Bertol.)	Patelliform	Patelliform	–	Anomocytic

### 2.3 Elemental analysis by SEM-EDAX

**Fig. 5:**
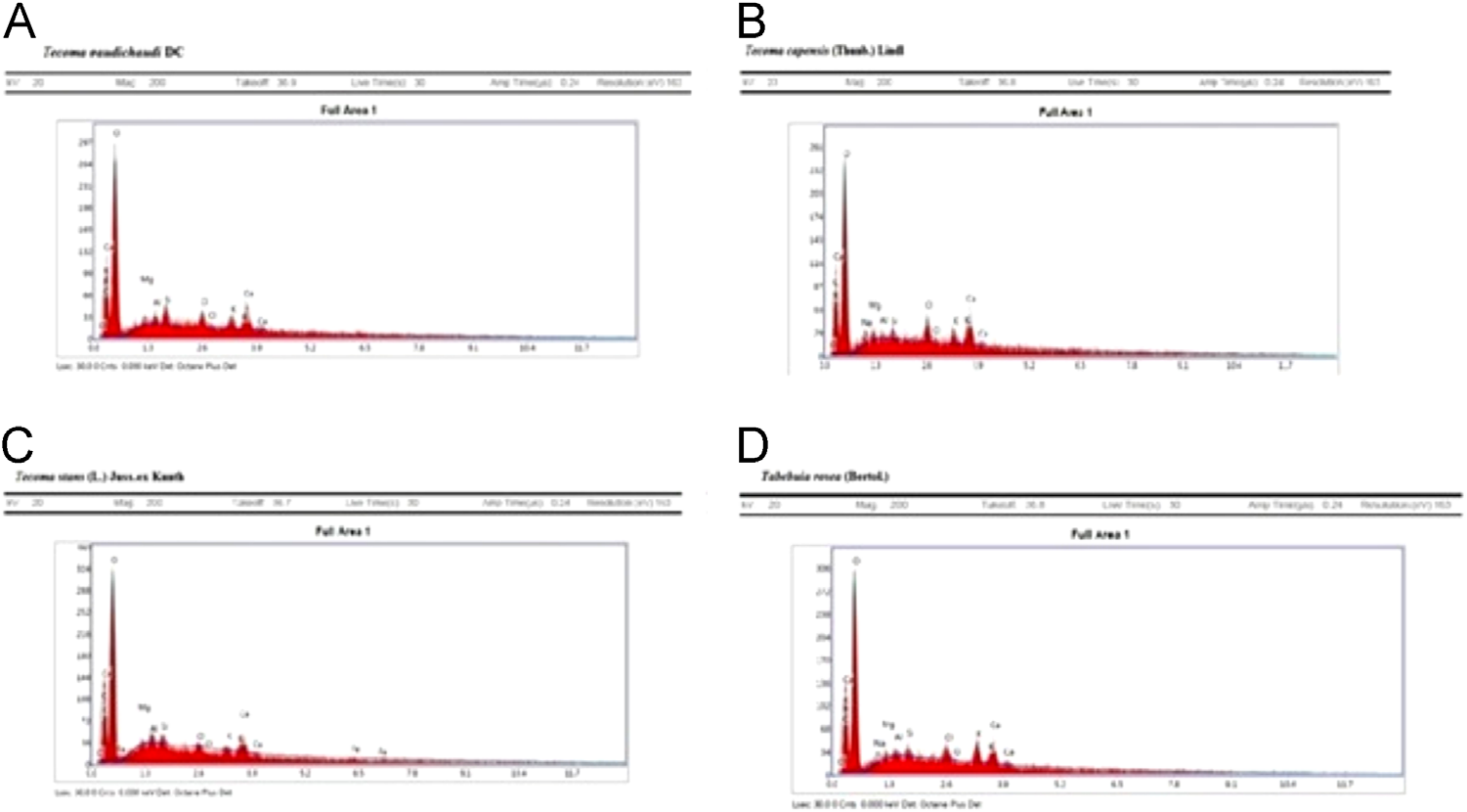
Elemental analysis of plant species by SEM-EDAX (A) Elemental profile of *Tecoma gaudichaudi* DC (B) *Tecoma capensis* (Thunb.) Lindl (C) *Tecoma stans* (L.) Juss.ex Kunth (D) *Tabebuis rosea* (Bertol).

Elemental data were performed by AMETEK EDAX (Model octane plus) with microscope/port QUANTA 200 ESEM/EDS. Present data on elemental analysis of all samples shows the following interpretation (Table 2 & Fig. 5).

**Table 2:** Elemental analysis of various species of Bignoniaceae Family.

Elements	Weight %			
	*Tecoma gaudichaudi* DC	*Tecoma capensis* (Thunb.) Lindl	*Tecoma stans* (L.) Juss.ex Kunth	*Tabebuia rosea* (Bertol.)
C	27.55	27.40	30.49	28.99
O	62.96	61.57	65.23	62.50
Na	–	2.47	–	1.70
Mg	1.04	0.87	0.27	0.42
Al	0.63	0.32	0.61	0.34
Si	1.28	0.58	0.40	0.50
Cl	1.55	1.98	0.27	1.10
K	1.61	1.53	0.34	1.93
Ca	3.38	3.27	2.22	2.53
Fe	–	–	0.17	–

The essential 8 elements were found to be in leaf sample of *Tecoma gaudichaudi* DC, *Tecoma capensis* (Thunb.) Lindl., *Tecoma stans* (L.) Juss.ex Kunth, *Tabebuia rosea* (Bertol.) such as C, O, Mg, Al, Si, Cl, K, Ca etc. The most important finding of this work was that, the leaves of *Tecoma gaudichaudi* DC showed a high concentration of all elements except Na and Fe. This type of study was reported first time on all these species, definitely help in selection of plant species for polyherbal formulation.
