# Supplementary material for: Dataset on leaf surface and elemental study of four species of Bignoniaceae family by SEM-EDAX
Source: Data Brief. 2018 Feb 17;17:1188–95. doi: 10.1016/j.dib.2018.02.037 (PMC5988445; doi:10.1016/j.dib.2018.02.037)
Supplement: Supplementary file 1 — Supplementary material. [file mmc1.pdf]

Comparative leaf surface study and elemental analysis of four species of Bignoniaceae family by SEM-EDAX.

Kalyani A. Kedar*[1], Sanjay R.Chaudhari[2], Avanapu S. Rao[3]

The Authors declared no conflict of Interest.

1. Corresponding author
Kedar Kalyani Abhimanyu
Assistant professor
Department of Pharmacognosy,
Progressive Education society's Modern College of Pharmacy,
Sector -21, Yamunanagar Nigdi, Pune-411044, Maharastra
Research scholar, Jawaharlal Nehru Technological University (JNTU),
Hyderabad, Andra Pradesh, India 500072
+91 9764012744 (m)
Fax no. 020-27661314.
E mail id: kk_pharma20@rediffmail .com

2. Dr. Chaudhari Sanjay Ravindra
Principal & Professor
Rasiklal M. Dhariwal Institute of Pharmaceutical Education and Research, Pune
ph:9822299601
e-mail: dr_srchaudhari@yahoo.com

3. Dr. Rao Avanapu Srinivasa
Principal & Professor
Bhaskar Pharmacy College,
Yeknapally, Moinabad(Mandal)
R.R(Dt), Hyderabad-500075
Ph: +91-9704014333(M)
E-Mail: dravanapu@yahoo.com
