# Supplementary material for: Dataset on leaf surface and elemental study of four species of Bignoniaceae family by SEM-EDAX
Source: Data Brief. 2018 Feb 17;17:1188–95. doi: 10.1016/j.dib.2018.02.037 (PMC5988445; doi:10.1016/j.dib.2018.02.037)
Supplement: Supplementary file 2 — Supplementary material [file mmc2.pdf]

PES Modern College of Pharmacy

Author: support
Creation: 3/25/2016

Sample Name: Tabebuia rosea

## Area 2

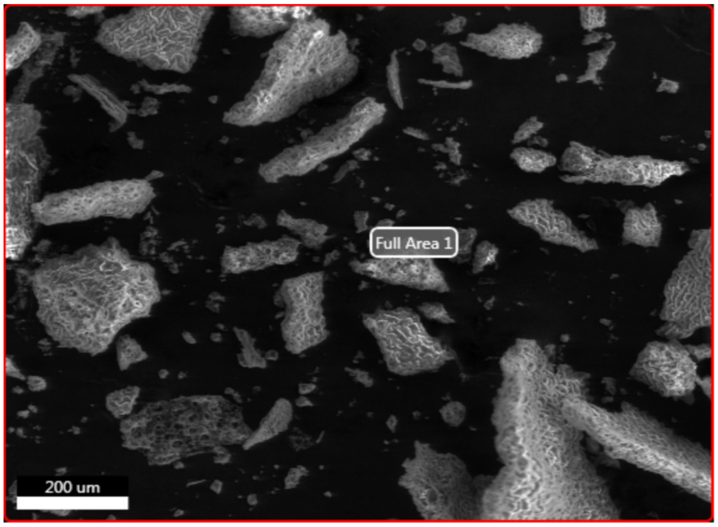


Notes:

## Full Area 1

| kV: 20 | Mag: 200 | Takeoff: 36.8 | Live Time(s): 30 | Amp Time(μs): 0.24 | Resolution:(eV) 163 |
|---|---|---|---|---|---|

**Full Area 1**

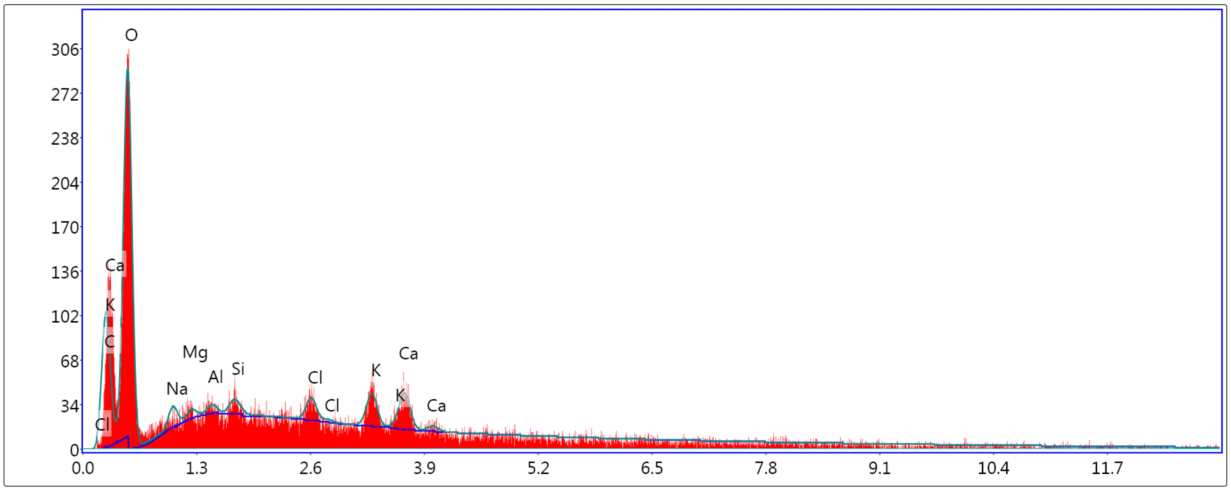


Lsec: 30.0 0 Cnts 0.000 keV Det: Octane Plus Det

**eZAF Smart Quant Results**

| Element | Weight % | Atomic % | Net Int. | Error % | Kratio | Z | R | A | F |
|---|---|---|---|---|---|---|---|---|---|
| C K | 28.99 | 36.65 | 75.16 | 99.99 | 0.13 | 1.04 | 0.98 | 0.42 | 1 |
| O K | 62.50 | 59.33 | 228.26 | 10.16 | 0.16 | 1 | 1 | 0.26 | 1 |
| NaK | 1.70 | 1.12 | 10.14 | 65.22 | 0.01 | 0.9 | 1.02 | 0.33 | 1 |
| MgK | 0.42 | 0.26 | 4.77 | 68.32 | 0.00 | 0.92 | 1.03 | 0.48 | 1 |
| AlK | 0.34 | 0.19 | 4.86 | 66.66 | 0.00 | 0.88 | 1.04 | 0.63 | 1 |
| SiK | 0.50 | 0.27 | 8.59 | 61.02 | 0.00 | 0.9 | 1.04 | 0.76 | 1 |
| ClK | 1.10 | 0.47 | 17.11 | 32.60 | 0.01 | 0.84 | 1.06 | 0.97 | 1.01 |
| K K | 1.93 | 0.75 | 25.54 | 20.44 | 0.02 | 0.84 | 1.07 | 1 | 1.01 |
| CaK | 2.53 | 0.96 | 28.03 | 19.20 | 0.02 | 0.85 | 1.07 | 1.01 | 1 |
