# Supplementary material for: Dataset on leaf surface and elemental study of four species of Bignoniaceae family by SEM-EDAX
Source: Data Brief. 2018 Feb 17;17:1188–95. doi: 10.1016/j.dib.2018.02.037 (PMC5988445; doi:10.1016/j.dib.2018.02.037)
Supplement: Supplementary file 3 — Supplementary material [file mmc3.pdf]

PES Modern College of Pharmacy

Author: support
Creation: 3/25/2016

Sample Name: Tecoma Capensis

Area 2

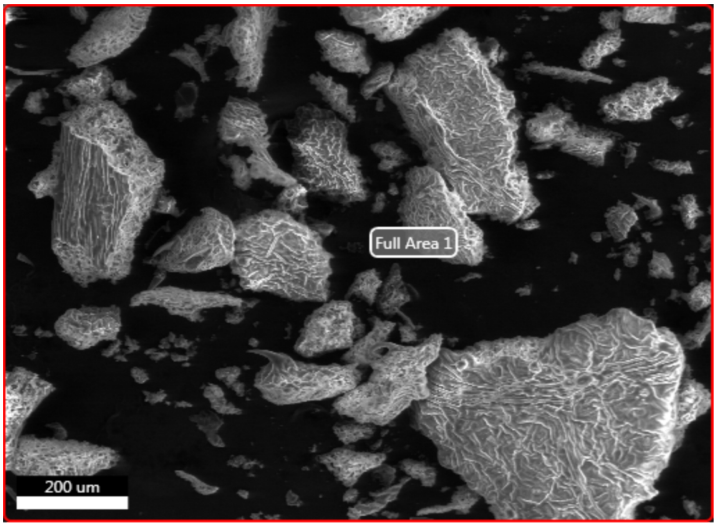

Notes:

Full Area 1

kV: 20	Mag: 200	Takeoff: 36.8	Live Time(s): 30	Amp Time(µs): 0.24	Resolution:(eV) 163

Full Area 1

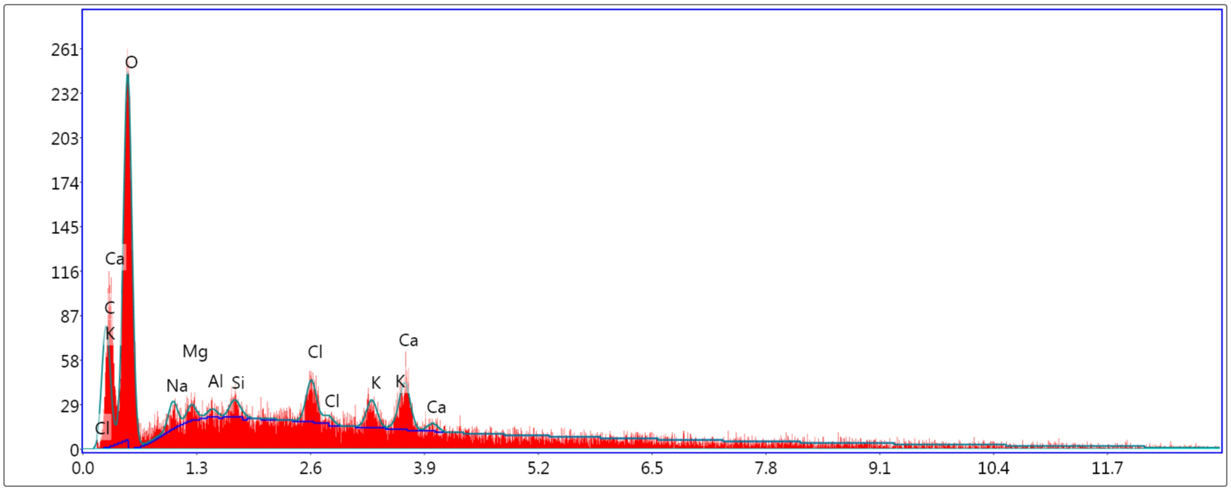

Lsec: 30.0 0 Cnts 0.000 keV Det: Octane Plus Det

eZAF Smart Quant Results

Element	Weight %	Atomic %	Net Int.	Error %	Kratio	Z	R	A	F
C K	27.40	35.19	54.68	99.99	0.11	1.05	0.98	0.37	1
O K	61.57	59.37	192.29	10.29	0.16	1	1	0.26	1
NaK	2.47	1.66	12.78	37.15	0.01	0.91	1.02	0.33	1
MgK	0.87	0.55	8.42	64.56	0.00	0.92	1.03	0.47	1
AlK	0.32	0.18	3.91	68.18	0.00	0.89	1.03	0.62	1
SiK	0.58	0.32	8.64	60.93	0.00	0.91	1.04	0.75	1
ClK	1.98	0.86	26.48	20.48	0.02	0.84	1.06	0.96	1.01
K K	1.53	0.61	17.45	26.29	0.01	0.84	1.07	1	1.02
CaK	3.27	1.26	31.08	18.03	0.03	0.86	1.07	1	1
