# Supplementary material for: Dataset on leaf surface and elemental study of four species of Bignoniaceae family by SEM-EDAX
Source: Data Brief. 2018 Feb 17;17:1188–95. doi: 10.1016/j.dib.2018.02.037 (PMC5988445; doi:10.1016/j.dib.2018.02.037)
Supplement: Supplementary file 4 — Supplementary material [file mmc4.pdf]

PES Modern College of Pharmacy

Author: support
Creation: 3/25/2016

Sample Name: Tecoma Gaudichaudi

Area 2

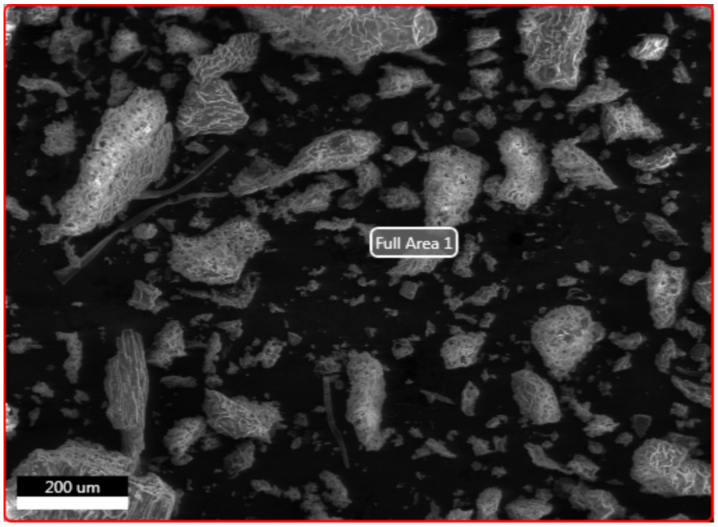

Notes:

Full Area 1

kV: 20	Mag: 200	Takeoff: 36.9	Live Time(s): 30	Amp Time(μs): 0.24	Resolution:(eV) 163

Full Area 1

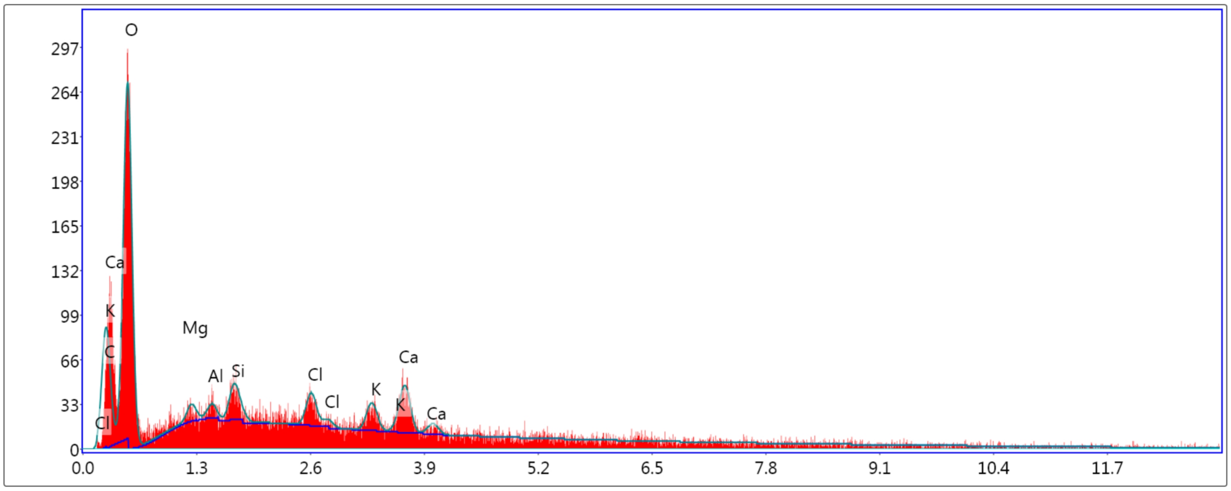

Lsec: 30.0 0 Cnts 0.000 keV Det: Octane Plus Det

eZAF Smart Quant Results

Element	Weight %	Atomic %	Net Int.	Error %	Kratio	Z	R	A	F
C K	27.55	35.24	61.54	99.99	0.11	1.04	0.98	0.38	1
O K	62.96	60.45	214.07	10.08	0.16	1	1	0.26	1
MgK	1.04	0.66	11.22	63.04	0.00	0.92	1.03	0.49	1
AlK	0.63	0.36	8.67	62.84	0.00	0.89	1.03	0.64	1
SiK	1.28	0.70	20.92	24.03	0.01	0.91	1.04	0.76	1
ClK	1.55	0.67	22.60	24.25	0.01	0.84	1.06	0.96	1.01
K K	1.61	0.63	19.99	22.16	0.01	0.84	1.07	1	1.02
CaK	3.38	1.29	35.11	18.55	0.03	0.85	1.07	1	1
