# Supplementary material for: Dataset on leaf surface and elemental study of four species of Bignoniaceae family by SEM-EDAX
Source: Data Brief. 2018 Feb 17;17:1188–95. doi: 10.1016/j.dib.2018.02.037 (PMC5988445; doi:10.1016/j.dib.2018.02.037)
Supplement: Supplementary file 5 — Supplementary material [file mmc5.pdf]

PES Modern College of Pharmacy

Author: support
Creation: 3/25/2016

Sample Name: Tecoma Stans

Area 2

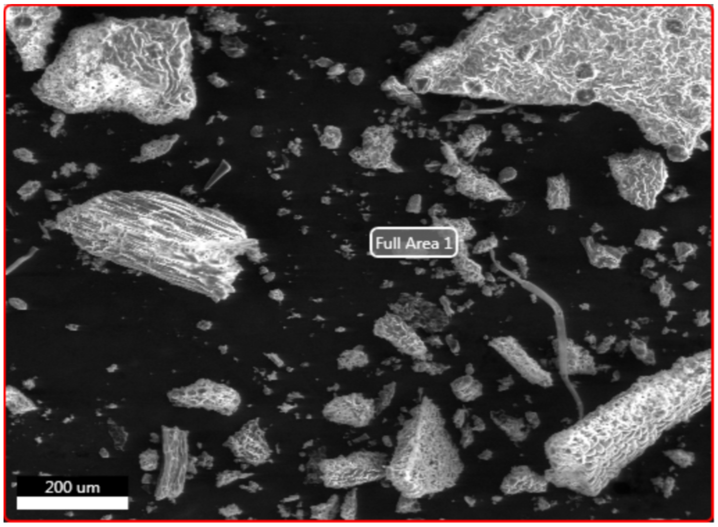

Notes:

Full Area 1

kV: 20	Mag: 200	Takeoff: 36.7	Live Time(s): 30	Amp Time(μs): 0.24	Resolution:(eV) 163

Full Area 1

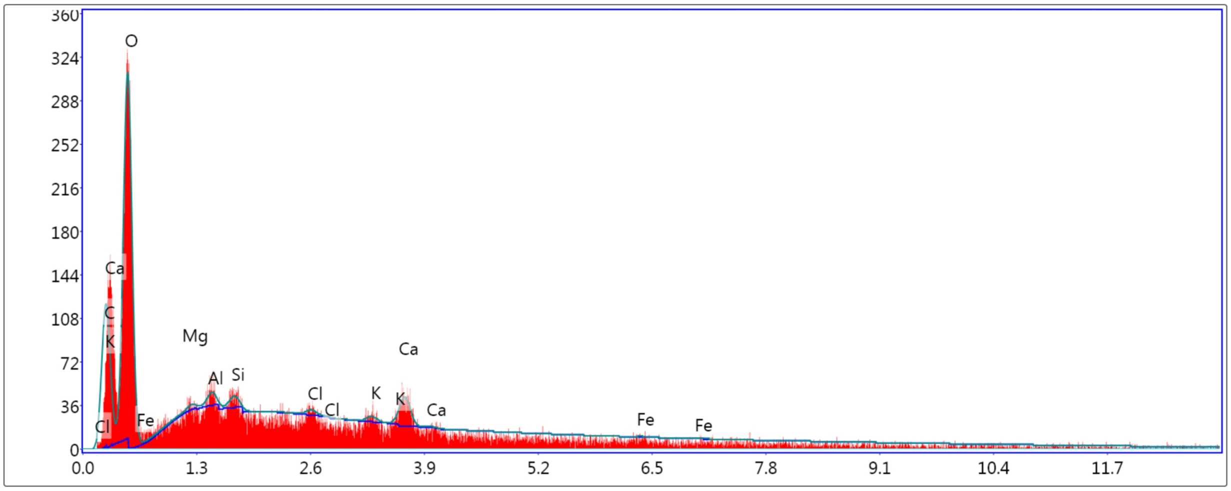

Lsec: 30.0 0 Cnts 0.000 keV Det: Octane Plus Det

eZAF Smart Quant Results

Element	Weight %	Atomic %	Net Int.	Error %	Kratio	Z	R	A	F
C K	30.49	37.67	83.36	99.99	0.15	1.04	0.98	0.46	1
O K	65.23	60.51	240.92	9.97	0.18	0.99	1	0.28	1
MgK	0.27	0.17	2.94	72.53	0.00	0.91	1.03	0.48	1
AlK	0.61	0.34	8.52	62.95	0.00	0.88	1.04	0.64	1
SiK	0.40	0.21	6.54	62.73	0.00	0.9	1.04	0.77	1
ClK	0.27	0.11	4.00	60.67	0.00	0.84	1.06	0.97	1.01
K K	0.34	0.13	4.30	60.23	0.00	0.83	1.07	1.01	1.01
CaK	2.22	0.82	23.57	21.52	0.02	0.85	1.07	1.02	1
FeK	0.17	0.05	0.84	67.44	0.00	0.75	1.09	1.02	1
